# Tibial Plasmablastic Lymphoma in a HIV-Negative Child: A Novel Extraoral Localisation

**DOI:** 10.1155/2022/4353830

**Published:** 2022-06-08

**Authors:** Ali Mala, Yameena Noman Khan, Akbar Ahmed, Naema Khayyam, Kanwal Aftab

**Affiliations:** Indus Hospital and Health Network, Plot C-76, Sector 31/5, Opposite Darussalam Society Korangi Crossing, Karachi 75190, Pakistan

## Abstract

Plasmablastic lymphoma is an aggressive, high-grade non-Hodgkin lymphoma predominantly seen in HIV-infected individuals. Alongside a strong correlation with HIV, PBL can manifest in immunocompromised HIV-negative patients. A rare case of PBL in an immunocompetent and otherwise healthy child presented to Indus Hospital & Health Network (IHHN), Karachi, Pakistan. The patient had complaints of swelling and pain in the right leg and was referred from a city in Interior Sindh. Histopathological analysis revealed sheets and aggregates of neoplasm replacing bone marrow interspersed with sclerotic bony fragments. Large, monomorphic, multinucleated neoplastic cells containing abundant cytoplasm and scattered pleomorphic cells were also noted, leading to the diagnosis of tibial plasmablastic lymphoma. A FAB/LMB96 group C chemotherapy regimen for aggressive and high-risk cancer was administered with a marked improvement in clinical symptoms.

## 1. Introduction

Plasmablastic lymphoma (PBL) is an aggressive, high-grade non-Hodgkin's lymphoma (NHL). Initially considered a variation of diffuse large B-cell lymphoma (DLBCL), PBL was later classified as a distinctive mature B-cell lymphoma and separated from the class of DLBCL by WHO [1]. PBL is predominantly seen in HIV-infected individuals with a preponderance of adults with children accounting for only a handful of cases [2].

Plasmablastic lymphoma was first reported in 1997, when a series of sixteen diffuse large B-cell lymphomas with unique immunohistochemistry were presented in HIV-positive patients with predominant involvement of the oral cavity [3]. Along with the oral cavity, it may also occur in sites including but not exclusive to skin, bone, genitourinary tract, nasal cavity and paranasal sinuses, CNS, liver, lungs, and orbits [4].

Herein, we report a rare case of HIV-negative plasmablastic lymphoma in a 9-year-old male child who presented with swelling and pain in the right leg.

## 2. Case Presentation

A 9-year-old male, resident of Larkana, presented to the ER of Indus Hospital with complaints of swelling and pain in his right leg. The patient reported trauma to the right leg 16 months prior after which he developed a swelling that gradually increased over time. It was associated with moderate pain, exacerbated on walking. An examination revealed a vitally stable child with a limping gait. A hard and nontender swelling was palpable in the middle of the right lower limb measuring 4-5 cm. The patient had received treatment for osteomyelitis after a presumptive diagnosis made by a primary care physician in Larkana a year back. Biopsy was conducted from the right tibia at a primary care hospital in Larkana four months prior to presenting at Indus Hospital, and histopathological analysis confirmed a diagnosis of plasmablastic lymphoma.

On admission at IHHN, staging workup and routine baseline investigations were conducted. Magnetic resonance imaging (MRI) of the primary site demonstrated lobulated abnormal signal intensity enhancing soft tissue mass arising from the medullary cavity of the right tibia. These soft tissue lesions had an exophytic component along the anterior and posterior aspect of the right tibia. The anterior lesion had an axial dimension of 3.7 cm × 2.5 cm with a craniocaudal diameter of 5.3 cm. The lesion arising from the posterior aspect had an axial dimension of 5.1 cm × 4.1 cm with a craniocaudal dimension of 5.7 cm. The posterior lesion showed gastrocnemius infiltration. The peroneal neurovascular bundle was encased and the popliteal fossa remained intact. Anteriorly, the lesion extended subcutaneously, showing postcontrast enhancement within the cutaneous and subcutaneous soft tissues. The anterior tibial neurovascular bundle was intact along with the absence of intraarticular extension of the lesion. No synchronous lesion was identified in fibula. Medullary bone involvement of the tibia was 10.7 cm in length.

Computed tomography (CT) neck, chest, and abdomen were negative for metastasis. A lumbar puncture was done and the CSF findings were unremarkable. Bilateral bone marrow biopsy exhibited trilineage hematopoiesis with absence of neoplastic cellular infiltration leading to categorization as stage 1, limited to the right tibia. Histopathology of the right tibial lesion was reviewed at IHHN, revealing sheets and aggregates of neoplastic cells replacing bone marrow interspersed with sclerotic bony fragments. Large, monomorphic multinucleated neoplastic cells containing abundant cytoplasm and scattered pleomorphic cells were also noted. Cells with plasmacytoid features were imparted from visible plasma cells. Immunohistochemical stains CD138, CD 38, VS38c, and MUM1 plasma cell markers were positive in neoplastic cells. Ki-67 was positive and expressed an increased proliferative index, approximately 98%. Pax 5 was negative in neoplastic cells. The B-cell markers CD20 and CD79a were negative. Leukocyte common antigen CD45, NK cell marker CD56, CD30, and Alk-1 and T-cell markers CD3 and CD5 were negative. Monoclonal pattern of distribution with kappa strong positive in neoplastic cells was noted on immunohistochemistry. The patient was found to be HIV and Hepatitis B and C negative on screening. Epstein–Barr encoding region (EBER) test was not conducted due to high cost of testing.

The patient received chemotherapeutic protocol FAB/LMB96 group C for aggressive and high-risk non-Hodgkin's lymphoma. Chemotherapeutic regimen involved first cycle of prephase COP (cyclophosphamide, vincristine, and prednisolone) followed by 2 cycles of induction chemotherapy COPADM (cyclophosphamide, vincristine, prednisone, adriamycin, and high-dose methotrexate) along with intrathecal chemotherapy administered over 3-week intervals. The patient proceeded with consolidation courses of chemotherapy and received 2 cycles of CYVE (cytarabine, etoposide) followed by maintenance cycles. A marked reduction in the size of the externally visible swelling in the right lower limb along with improvement in gait and pain was noted via clinical assessment conducted over the course of 4 months. MRI scan posttreatment demonstrated soft tissue anterior compartment lesion measuring 0.8 cm anterior posterior ×0.5 cm transverse ×3.7 cm craniocaudal. These findings hence showed a marked reduction in the size of the lesion posttreatment. Medullary bone involvement was 10.4 cm in length. The posterior compartment lesion was not visible. These findings hence showed a marked reduction in the size of the lesion posttreatment. Following maintenance chemotherapy, positron emission tomography computed tomography (PET-CT) of skeletal system revealed nonfluorodeoxyglucose (FDG) avid irregular cortical sclerosis with multiple small breaks present in the mid two thirds of the shaft of the right tibia rated at 1 on a Deauville 5-point PET-CT scale. The size of sclerotic areas in the tibia was not available. No evidence of hypermetabolic lesions was seen in the skeletal system. Subcentimeter level II lymph nodes were noted with SUV max ranging up to 1.62, appearing infective. Hence, it is concluded that the patient is currently in remission and is being monitored clinically for any signs of recurrence. The patient experienced chemotherapy-related toxicities including mucositis, nausea, vomiting, and febrile neutropenia which were treated with antibiotics, filgrastim, and supportive care. Chemotherapy-induced myelosuppression causes anemia and thrombocytopenia leading to perirectal bleeding requiring blood transfusions in the form of packed red blood cells and platelets.

## 3. Discussion

Plasmablastic lymphoma is an aggressive B-cell malignancy that is recognized as a subtype of non-Hodgkin's lymphoma. It is highly associated with human immunodeficiency virus (HIV) and its estimated incidence accounts for approximately 5% of all HIV-positive NHL cases. Regardless of its strong relationship with HIV, PBL can also manifest in HIV-negative patients; however, such patients are presumed to have history of autoimmune or lymphoproliferative disorders [5]. This entity not only occurs in the clinical setting of HIV/AIDS but has also been reported in cases of latent Epstein–Barr virus (EBV) infections [6]. Even though features of PBL in an HIV-negative individual have previously been reported, the etiology of HIV-negative PBL is still uncertain. According to a comprehensive analysis, HIV-negative PBL occurs in a wide spectrum of patients with average age at the time of diagnosis found to be 58.9 years with a range of (2–86) years old. Moreover, the statistical data shows male predominance with male: female (2.29 : 1) occurring more in elderly patients (greater than 60 years old, 56.14%) [7]. Another report of 8 cases showed the mean age to be 53.0 years with a range of (27.0–69.0) years [8].

PBL almost as a rule presents as a mass in extranodal regions of the head and neck, in particular the oral cavity and only >1% of cases of extranodal localisations are reported [4]. However, recently, PBL has been discovered to invade extraoral regions which is supported by a number of cases, including a case of vulvar plasmablastic lymphoma in a HIV-positive child [9] and a cutaneous presentation of plasmablastic lymphoma in an EBV positive child [10]. Furthermore, there have been PBL reports on immunocompetent and HIV-negative individuals with lesions of the stomach and anorectal junction, respectively [11, 12].

In this case, the patient tested negative for HIV but was not tested for EBV due to its high cost and unavailability at IHHN.

The cell from which the tumour originates is itself an active, postgerminal centre B-lymphocyte which could be a likely reason for its plasma cell traits and plasmacytic differentiation markers. Such a distinct set of characteristics of PBL poses a diagnostic challenge and requires a strong observation for its recognition [13].

Since PBL is described by an immunoblastic or plasmablastic morphology and a plasma cell immunophenotype, its separation from other large B-cell lymphoma or plasmablastic plasma cell neoplasm is often difficult [14]. Hence, the articulation of immunohistochemical markers is an imperative component of the diagnostic protocol [15]. Plasmablastic lymphoma is diagnosed on the basis of specific histopathological features consisting of cohesive cellular proliferation of large atypical cells resembling immunoblasts with plasmacytic differentiation [4]. The immunohistochemical panel of these neoplastic cells expresses a plasma cell phenotype along with positivity for CD138, CD38, VS38c, IRF4/MUM1, PRDM1 (also called BLIMP1), and XBP1. However, CD45, CD20, and PAX5 appear to be either negative or sometimes weakly positive in a minority of cells. Moreover, cytoplasmic immunoglobulin is commonly expressed with the Ki-67 proliferation index being very high (>90%) [4]. It was demonstrated in this case an immunohistochemical profile that mirrored plasmablastic lymphoma containing CD138 (+) plasma cells and scattered linear recticulin with no intersections. The Ki-67 antigen exhibited an increased proliferative index of approximately 98%. CD20 and CD3 were found to be negative in neoplastic cells, indicating the presence of mature B and T lymphocytes. Kappa and lambda were positive and exhibited a monoclonal pattern along with negative CD79a.

The recent parameters for the treatment of lymphoma in initial stages include CHOP or similar chemotherapy regimens [11]. However, the treatment is not fixed and varies according to the case presentation. Chemotherapy using prednisolone, cyclophosphamide, adriamycin, and/or vincristine with or without field radiation therapy can be considered. However, the implementation of chemotherapy, radiotherapy, or a merger of both is observed in 38%, 27%, and 22% of cases, respectively. Some researchers have utilised highly active antiretroviral drugs in HIV-related PBL with a response rate of 14% [15]. Prognosis is generally poor in cases of PBL with patients surviving less than 12 months. Nonetheless, there are case reports documenting longer survival rates of more than 5–6 years in adults [16] (see Table 1).

The reported patient was treated with cyclophosphamide, vincristine, daunorubicin, and high-dose methotrexate. He received five cycles of chemotherapy, which showed a substantial decrease in lesion size on CT scans and of the externally visible swelling along with improvement in gait. Our patient belonged to a low socioeconomic background with decreased access to quality healthcare, a likely reason for the initial misdiagnosis of osteomyelitis and delay in diagnosis and treatment. Delay in seeking medical care, disorganised referral systems, and lack of specialists are other major factors in the lack of cases reported overall. Due to the unique histological nature and clinical presentation of plasmablastic lymphoma, there is a need for further research on diagnostic protocols and treatment trials along with the identification of predisposing factors such as causative genetic mutations and alterations.

**Figure 1:**
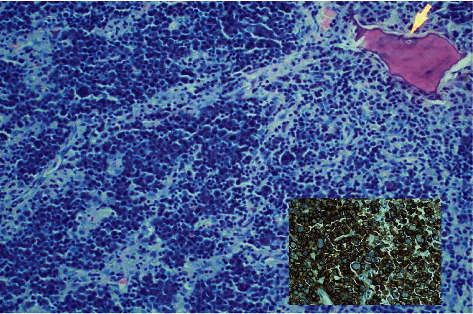
Photomicrograph showing sheets of tumor cells in the intertrabecular areas (hematoxylin and eosin stain, 400x). Inset shows strong positivity with CD138 (immunohistochemical stain, 400x).

**Figure 2:**
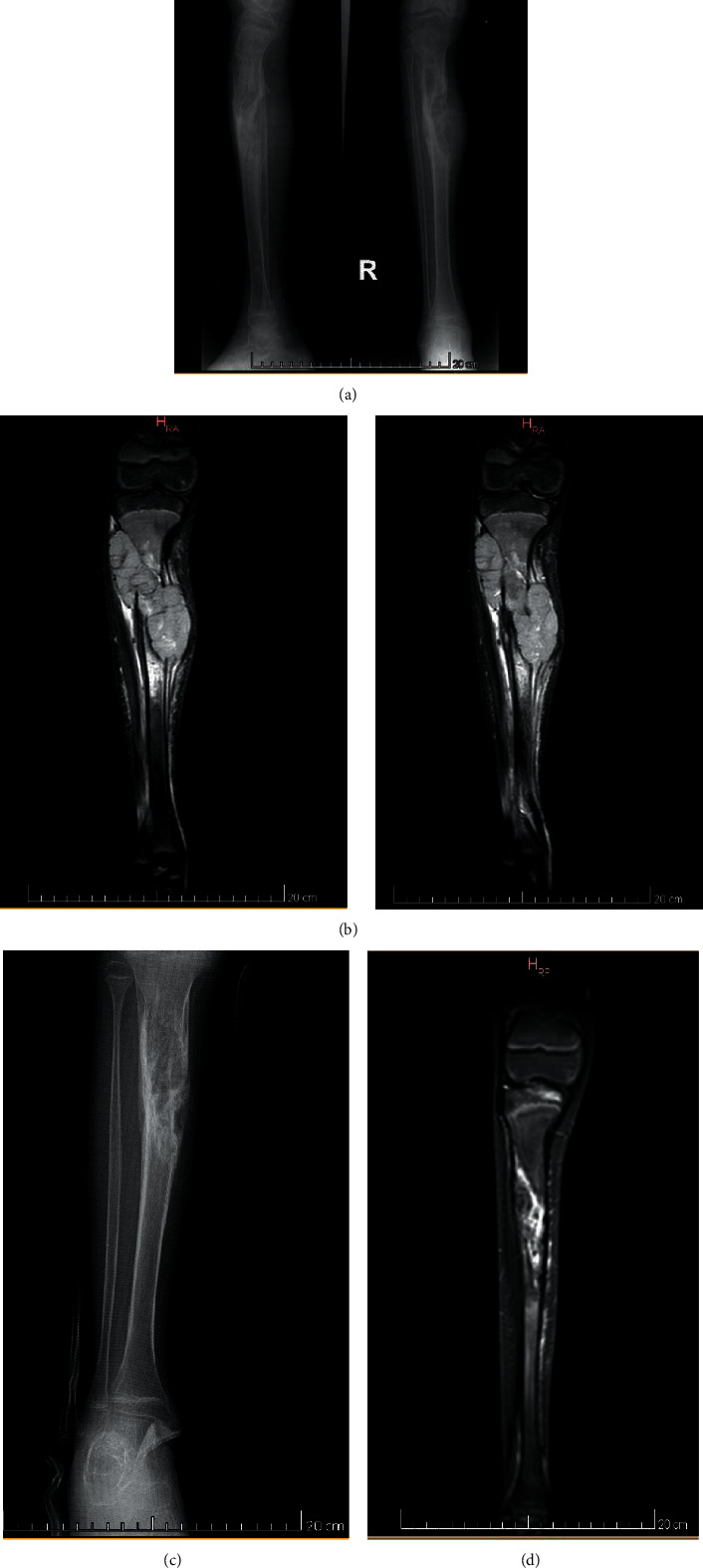
(a) X-ray at diagnosis, (b) MRI pretreatment, (c) X-ray posttreatment, and (d) MRI posttreatment.

**Figure 3:**
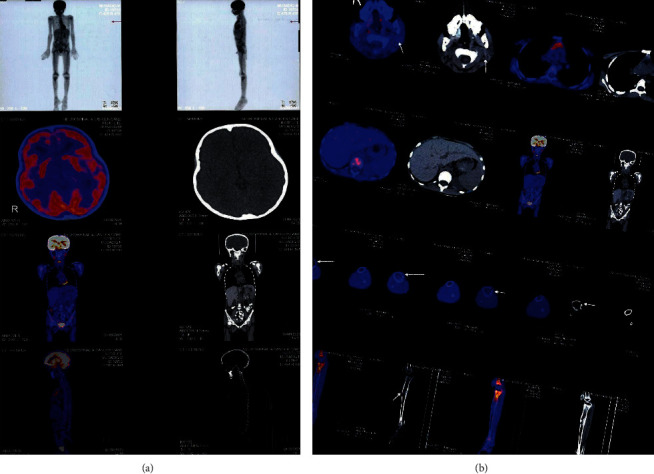
(a) PET-CT posttreatment and (b) PET-CT posttreatment.

An article in the American Journal of Hematology recorded a satisfactory initial response to therapy in 113 cases of both genders and a wide age group, but the rate of recurrence was high with the prognosis subpar. Components such as sex, lymphoma stage, primary site of involvement, CD4+ count, viral load, EBV status, and use of CHOP do not appear to foretell the survival rate in HIV-associated PBL. Improved and vigorous therapy regimes are suggested for future cases [17]. Another study also concurred with the low incidence of PBL and no appropriate protocol for this special subtype of NHL. This emphasises the need for newer therapies independent of HIV status [18] (see Figures 1–3).

## 4. Conclusion

Plasmablastic lymphoma is a NHL that most commonly arises in HIV-positive adults. Its diagnosis poses a serious challenge due to its similarities with DLBCL; hence, performing detailed immunophenotypic studies for establishing the diagnosis of PBL is pertinent. Furthermore, it is an aggressive malignancy with a relatively poor outcome and therefore requires a belligerent multimodality treatment.

## Figures and Tables

**Table 1:** Panel of immunohistochemical markers performed and results are as follows.

CD138	Diffuse strong positive in neoplastic cells

CD38	Positive in few neoplastic cells
CD56	Negative in neoplastic cells
Ki-67	Increased proliferative index approximately 98%
Kappa and lambda	Monoclonal pattern of distribution with kappa strong positive in neoplastic cells
CD20	Negative in neoplastic cells. Highlight mature B-lymphocytes in background
CD79a	Negative in neoplastic cells
PAX-5	Negative in neoplastic cells
CD30	Negative in neoplastic cells
Alk-1	Negative in neoplastic cells
CD3	Negative in neoplastic cells. Highlight mature T-lymphocytes in background.
CD45	Negative in neoplastic cells
CD5	Negative in neoplastic cells
